# Methods for Biomimetic Mineralisation of Human Enamel: A Systematic Review

**DOI:** 10.3390/ma8062873

**Published:** 2015-05-26

**Authors:** Chris Ying Cao, May Lei Mei, Quan-li Li, Edward Chin Man Lo, Chun Hung Chu

**Affiliations:** 1 Faculty of Dentistry, the University of Hong Kong, Hong Kong, China; E-Mails: caoying0713@gmail.com (C.Y.C.); modelbaby1981@gmail.com (M.L.M.); hrdplcm@hku.hk (E.C.M.L.); 2 Stomatological Hospital and College, Anhui Medical University, Key Lab. of Oral Diseases Research of Anhui Province, Hefei 230032, China; E-Mail: u3001279@connect.hku.hk

**Keywords:** biomimetic, mineralisation, enamel, proteins

## Abstract

Biomimetic mineralisation is an alternative restorative methodology that imitates the natural process of mineralisation. We aimed to systematically review the laboratory methods on the biomimetic mineralisation of demineralised enamel. A search in the PubMed, ScienceDirect, and ISI Web of Science databases was performed. Clinical trials, reviews, non-English articles, animal teeth, non-tooth substrates, and irrelevant studies were excluded. After screening the titles and abstracts of initially searched articles, 20 papers remained for full-text analysis. Eight articles were identified from the references of the remaining papers. A total of 28 studies were included in this systematic review. We found that protein or protein analogues were used to mimic the function of natural protein in 23 studies. Bioactive components inspired by mussel, an agarose hydrogel model, a glycerine-enriched gelatine technique, and ethylenediaminetetraacetic acid, were also used for biomimetic mineralisation of enamel. These laboratory studies reported success in the biomimetic mineralisation of enamel. Potential further research on the biomimetic mineralisation of enamel was discussed.

## 1. Introduction

Unlike the other mineralised tissues of the human body (*i.e.*, bone, dentine, and cementum), mature enamel is acellular and has more than 95% mineral content and less than 1% organic material, thus, making it the hardest mineralised tissue in the human body [1]. The crystals found in enamel are nanorod-like hydroxyapatite (HAP) crystals with hierarchical levels of the enamel microstructure from the nanoscale to microscale [2]. The highly organised hierarchical microstructure (prism) provides dental enamel its unique strength and anti-abrasive properties [3]. During enamel biomineralisation, ameloblasts secrete proteins, such as amelogenin and ameloblastin, to mediate the crystallites to form the well-organised prism pattern [4]. Although the initial formation of enamel apatite occurs under a protein-rich gel-like matrix environment, the biological macromolecules are almost degraded or removed gradually. This makes mature enamel a non-living tissue. Damaged enamel is permanent and does not remodel. As the outermost layer covering the tooth, enamel is susceptible to dental caries and erosive wear. Traditional treatments replace the defective enamel with dental restorative materials, such as amalgam, composite resin, and ceramics, however, these restorative materials are very different from natural enamel in chemical components, crystal structures, and physicochemical properties. They cannot fit well with enamel at the interface of the enamel, and the restorative materials are at risk of marginal leakage, which leads to hypersensitivity and secondary caries. Therefore, it is of great interest for scientists in different fields to design an alternative strategy to repair defective enamel.

Enamel is relatively stable in a healthy oral environment with the presence of saliva. The demineralisation and remineralisation of enamel are continuously taking place at the tooth-pellicle-plaque-saliva interface [5]. It is well established that dental caries is a dynamic disease process caused by the imbalance between demineralisation and remineralisation [6]. This concept of dental caries provides the scope for remineralising early enamel carious lesions. Recently, various remineralising strategies have been developed to restore early enamel lesions and prevent enamel demineralisation, such as fluoride [7,8], surfactants [9], electrolytic deposition [10], hydrothermal methods [1,10], and hydrogen peroxide [11]. However, these methods are performed under stringent conditions, such as extremely low acidity or high electric field environments, which cannot be translated to clinical applications. Although surfactants, such as reverse micelles, mimic the natural biomineralisation process of enamel by modifying the HAP nanorods to self-assemble into an enamel-like structure, the biocompatibility limits their clinical application [12]. Dental enamel is produced by ameloblasts. Enamel formation is a protein-mediated biomineralisation process that takes place within an extracellular matrix [4,13,14]. Amelogenin is the major component of this matrix and it is essential for proper enamel biomineralisation [15]. Biomimetic mineralisation is a methodology that imitates the natural process of mineralisation [16]. It is generally accepted that the biomimetic synthesis of enamel-like apatite structures under a physiological condition is an alternative restorative pathway. This paper is a systematic review of the methods that successfully achieved the biomimetic mineralisation of demineralised human enamel.

## 2. Results

**Figure 1:**
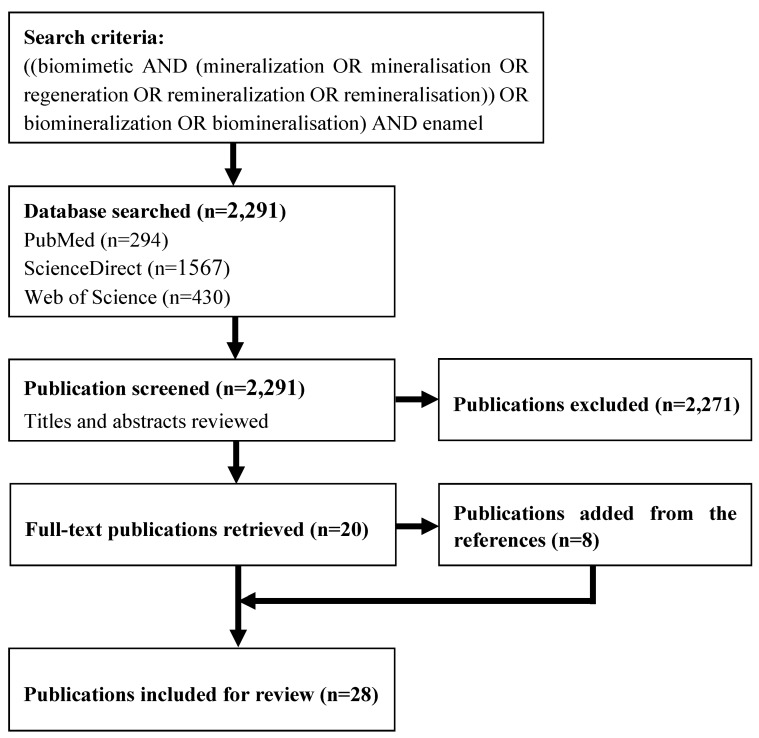
Flowchart of the search strategy.

The initial search from three electronic databases identified 2291 potential articles (294 in PubMed, 1567 in ScienceDirect, and 430 in ISI Web of Science), 2271 were excluded after the initial screening of titles and abstracts. After retrieving the full text of the remaining 20 articles, eight articles were found from the bibliographies of the included articles. Therefore, 28 articles were included in the systematic review (Figure 1). All 28 articles were *in vitro* studies published (including those Epub ahead of print) between 1997 and 2014.

Table 1 summarised the different methods of biomimetic mineralisation of enamel used in the included articles. Protein or protein analogues were used to mimic the function of natural proteins for the biomimetic mineralisation of enamel in 23 studies. Two studies used bioactive components inspired by mussel as a template to facilitate the hydroxyapatite nucleation on enamel [17,18]. A study constructed an agarose hydrogel model for the biomimetic mineralisation of enamel [19]. A study developed a glycerine-enriched gelatine technique to form fluorapatite layers on enamel [20]. A study used ethylenediaminetetraacetic acid (EDTA) as the mediating agent to reconstruct fluoride hydroxyapatite on enamel [21].

Mineral ions were provided by different remineralising mediums in the included articles. The most commonly used remineralising medium was artificial saliva. In addition, simulated body fluid (SBF), calcium phosphate remineralising solution/hydrogel, apatite nanocrystals slurry, bioactive glass slurry, and human saliva were also used. Fluoride at different concentrations was added in the remineralising mediums in 15 studies. The highest concentration of fluoride was 1100 ppm and the lowest concentration was 0.45 ppm.

Different demineralising agents were used to treat enamel surface before biomimetic mineralisation. Phosphoric acid at concentration from 30% to 37% was used in 17 studies. In addition, a demineralising solution [22,23,24,25,26], citric acid [27], nitric acid [28,29,30], acidified gelatine gel [31], and a bi-layer demineralisation protocol of methylcellulose gel buffered with a lactic acid layer [32] were also used. In fact, the different demineralization agents are used to create two different models of enamel defects. The strong acid (phosphoric acid or nitric acid) is used to create an erosive lesion, while the demineralising solutions (or some gels) usually with pH of ~4.6 are used to generate artificial caries. These studies can regenerate enamel-like crystals on the enamel surface, and the main findings can be found in Table 1.

The functions of the proteins or protein analogues are summarised in Table 2. Amelogenin was added into a calcium phosphate solution containing fluoride [28,29] or mixed with hydrogel containing calcium and phosphate [26,33,34] to promote the orderly enamel-like structure formation. In one study, an enamel matrix derivative was mixed with calcium chloride agarose hydrogel to guide the continuous growth of crystals, prevent the crystal fusion of premature crystals, and control the nucleation and growth of the enamel prism-like crystals [35]. A polyamidoamine (PAMAM) dendrimer solution was applied to the etched enamel surface to provide nucleation sites and a mineralisation template for nanorod-like HAP formation with high uniformity [30,36,37]. Peptide was the most commonly used protein analogue. The sequence of the different peptides used in the included articles was summarised in Table 2. Only one study added peptide into a calcium phosphate solution containing fluoride to induce the formation of stable amorphous calcium phosphate (ACP) nano-precursors and prevent them from aggregation and precipitation [38]. In the other seven studies, a peptide solution was applied to the etched enamel surface to bind with the HAP surface, capture calcium and phosphate ions from artificial saliva, and promote HAP with a small average crystalline size [27,31,39,40,41,42,43]. Glutamic acid (Glu) was added into SBF to induce nano-apatite particle assembly and aggregation on the etched enamel surface, resulting in the regeneration of the enamel-like structure [44]. Polyacrylic acid (PAA) was added to bioactive glass and applied to the etched enamel surface to promote the oriented bundle formation, release Ca and P ions to form an ACP layer, and act as a reservoir of ions available for remineralisation [32]. The nano-complexes of the phosphorylated chitosan-ACP solution were applied to the etched enamel surface to act as the aggregates of prenucleation clusters, to provide stable ACP, and to adsorb on the enamel surface [25]. Etched enamel was incubated with a casein phosphopeptide-amorphous calcium phosphate (CPP-ACP) solution [22], coated with CPP-ACP paste [23], or brushed with CPP-ACP paste [24] to act as a calcium and phosphate reservoir and promote enamel subsurface lesion remineralisation.

**Table 1:** Summary of the *in vitro* studies on biomimetic mineralisation on human enamel.

Authors, Year [Ref.]	Surface Treatment	Biomineralisation Method	Sources of Ca and P	Main Finding
Reynolds, 1997 [22]	Demineralising solution (4d)	CPP-ACP	CaCl_2_ solution, K_3_PO_4_ buffer	The CPP-stabilised CaP solution can remineralise subsurface enamel lesions.
Busch, 2004 [20]	30% PA (30s)	Glycerin-enriched gelatin 620 ppm F	CaCl_2_ solution, PO_4_ gelatin	Ordered enamel-like mineral was induced on human teeth.
Kirkham *et al.*, 2007 [31]	Acidified gelatine gel (6w)	Peptide	pH cycling	Peptide treatment increased remineralisation and inhibited demineralisation.
Kumar *et al.*, 2008 [23]	Demineralising solution (4d)	CPP-ACP 1100 ppm F	pH cycling	CPP-ACP remineralised initial enamel lesions.
Fan *et al.*, 2009 [28]	3% HNO_3_ solution (50s)	Amelogenin 0.45 ppm F	Calcification solution	The synthetic nanorod crystals formed on etched enamel in the presence of amelogenin.
Xie *et al.*, 2011 [21]	37% PA (30s)	EDTA 950 ppm F	Ca-EDTA complex-phosphate solution	EDTA induced the assembly of hexagonal prism-like FHAP microcrystals.
Jayarajan *et al.*, 2011 [24]	Demineralising solution (5h)	CPP-ACP, CPP-ACPF 900 ppm	Artificial saliva	CPP-ACPF showed more amount of remineralisation than CPP-ACP.
Fan *et al.*, 2011 [29]	5% HNO_3_ solution (30s)	Amelogenin 1–10 ppm F	Remineralisation solution	Densely packed arrays of FHAP nanorods were observed.
Hsu *et al.*, 2011 [39]	35% PA (30s)	Peptide	SBF	The nanomechanical properties of the acid-demineralised enamel were greatly improved.
Hsu *et al.*, 2011 [42]	35% PA (30s)	Peptide	SBF	Peptide promoted the uniform deposition of nano-crystalline CaP over enamel surfaces.
Li *et al.*, 2011 [44]	37% PA (10s)	Glu-apatite nanoparticles	SBF	Nanoparticles turned into rod-like crystals on enamel surface.
Zhou *et al.*, 2012 [17]	37% PA (2m)	Polydopamine 1 ppm F	Calcification solution	No significant difference was observed in the remineralisation of enamel, except in dentine.
Fan *et al.*, 2012 [26]	Demineralising solution (3d)	Amelogenin 5 ppm F	Artificial saliva	Application of the amelogenin-hydrogel significantly improved enamel hardness.
Chung *et al.*, 2012 [40]	34% PA (15s)	Peptide	Artificial saliva	Peptide promoted the uniform deposition of apatites with small crystalline size.
Li *et al.*, 2013 [18]	37% PA (15s)	Nacre water-soluble matrix	SBF	Water-soluble matrix facilitated the formation of HAP nanorods.
Chung *et al.*, 2013 [27]	1M citric acid (2m)	Peptide	Artificial saliva	Peptide promoted the formation of nano-HAP crystals.
Chen *et al.*, 2013 [30]	3% HNO_3_ solution (50s)	PAMAM 1 ppm F	Calcification solution	PAMAM could induce the formation of HAP crystals on demineralised enamel.
Ruan *et al.*, 2013 [33]	30% PA (30s)	Amelogenin 136 ppm F	Amelogenin-CaP hydrogel, artificial saliva	The enamel-like layer was formed on natural enamel.
Wu *et al*., 2013 [36]	37% PA (45s)	PAMAM	Artificial saliva	PAMAM could induce nanorod-like HAP remineralisation on acid-etched enamel.
Chung *et al.*, 2013 [41]	34% PA (30s)	Peptide	Artificial saliva	Peptide attracted ions from artificial saliva to form HAP crystals and fill enamel caries.
Chung *et al.*, 2013 [43]	34% PA (30s)	Peptide	Artificial saliva	Peptide promoted the deposition of small-grain HAP crystals.
Cao *et al.*, 2014 [19]	37% PA (60s)	Agarose hydrogel 500 ppm F	CaCl_2_ hydrogel, Phosphate solution	Enamel prism-like tissue was generated on etched enamel surface.
Zhang *et al.*, 2014 [25]	Demineralising solution (7d)	Phosphorylated chitosan-ACP	Pchi-ACP solution	Remineralising rate of Pchi-ACP treatment was higher than that of fluoride treatment.
Ruan *et al.*, 2014 [34]	30% PA (30s)	Amelogenin 0.45 ppm F	Amelogenin-CaP hydrogel, artificial saliva	Enamel-like organized apatite crystals can be formed in amelogenin-chitosan system.
Milly *et al.*, 2014 [32]	8% methylcellulose gel, lactic acid (14d)	Polyacrylic acid, Bioactive glass	Bioactive glass slurry	BAG and PAA-BAG treatments enhanced remineralisation of enamel white spot lesions.
Cao *et al.*, 2014 [35]	37% PA (60s)	Enamel matrix derivative 500 ppm F	EMD-CaCl_2_ hydrogel, Phosphate solution	EMD facilitated enamel prism-like tissue formation on demineralised human enamel.
Chen *et al.*, 2014 [37]	37% PA (45s)	PAMAM	Artificial saliva	PAMAM could produce an enamel prism-like structure on acid-etched enamel.
Li *et al.*, 2014 [38]	37% PA (60s)	Oligopeptide 1 ppm F	Metastable calcium phosphate solution	Oligopeptide induced the formation of HAP crystals on etched enamel.

BAG—Bioactive glass; CPP—Casein phosphopeptide; CPP-ACP—Casein phosphopeptide-amorphous calcium phosphate; CPP-ACPF—Casein phosphopeptide-amorphous calcium phosphate fluoride; EDTA—Ethylenediamine tetraacetic acid; EMD—Enamel matrix derivative; F—fluoride; FHAP—Fluoridated hydroxyapatite; Glu—glutamic acid; HAP—Hydroxyapatite; PA—Phosphoric acid; Pchi—Phosphorylated chitosan; PAA—Polyacrylic acid; PAMAM—Poly (amido amine) dendrimer; PVP—poly(vinylpyrrolidone); SBF—Simulated body fluid.

**Table 2:** Protein or its analogues and their functions in biomimetic mineralisation of human enamel.

Protein or Its Analogues	Function of Protein or Protein Analogues	Approach [Ref.]
Amelogenin	Promote the oriented bundle formationMediate the orderly enamel-like structure formation Mediate the orderly enamel-like structure formation Control the organised growth of apatite crystals Form transitional complexes with ACP Promote the hydrolysis of ACP to stable HAP Stabilise the Ca-P clusters and guide their arrangement into linear chains	Amel—Ca/P/F solution: [28,29] Amel—Ca/P hydrogel: [26,33,34]
EMD	Affect the formation of enamel prism-like tissue Guide the continuous growth of crystals Prevent the crystal fusion of premature crystals Control crystal morphology and subsequent elongation Control the nucleation and growth of the crystals	EMD—Ca agarose hydrogel: [35]
PAMAM dendrimers: PAMAM-COOH [30] ALN-PAMAM-COOH [36] PAMAM-PO_3_H_2_ [37]	Absorb onto the etched enamel surface Provide nucleation sites and mineralisation template for HAP Regulate the growth of crystals Induce nanorod-like HAP with high uniformity Induce *in situ* remineralisation of HAP on etched enamel	PAMAM—etched enamel: [30,36,37]
Peptide: 3NSS [27,41,43] QQRFEWEFEQQ [31] C_18_H_35_O-TKREEVD [38] 8DSS [39,42] 3DSS [40]	Form 3D biomimetic scaffolds capable of nucleating HAP Capture Ca and P ions from artificial saliva Limit Ca and P ions to depart from the demineralised enamel Possess a high affinity to bind with the HAP surface Promote HAP with a small average crystalline size Induce formation of stable ACP nano-precursors and prevent them from aggregation and precipitation	Peptide—etched enamel: [27,31,39, 40, 41, 42, 43] Peptide—Ca/P/F solution: [38]
Glutamic acid	Induce the nano apatite assembly on enamel Provide the active sites to control enamel formation Interact with a (001) surface of the nano apatite Induce the oriented aggregation of nano apatite	Glutamic acid—SBF: [44]
Polyacrylic acid	Sequester calcium and phosphate ions Form amorphous calcium phosphate nano-precursors Reduce the abrasiveness of bioactive glass particles	Polyacrylic acid—bioactive glass: [32]
Phosphorylated chitosan	Bind calcium ions to form nucleating sites Stabilise ACP to form the nano-complexes of Pchi-ACP Adsorb onto the surfaces of HAP crystals Inhibit spontaneous precipitation of calcium phosphate	Pchi—CaPO_4_ solution: [25]
Casein phosphopeptide	Stabilise calcium, phosphate, and hydroxide ions Prevent spontaneous precipitation of calcium phosphate Promote enamel subsurface lesion remineralisation	CPP—CaPO_4_ solution: [22] CPP—ACP Paste: [23,24]

ACP—Amorphous Calcium Phosphate; ALN—Alendronate; Amel—Amelogenin; Ca—Calcium; CPP—Casein Phosphopeptide; DSS—Aspartic acid-Serine-Serine; EMD—Enamel Matrix Derivative; F—Fluoride; HAP—Hydroxyapatite; NSS—Asparagine-Serine-Serine; P—Phosphorus; PAMAM—Poly (Amido Amine) Dendrimer; Pchi—Phosphorylated Chitosan; QQRFEWEFEQQ—Glutamine-Glutamine-Arginine-Phenylalanine-Glutamic acid-Tryptophan-Glutamic acid-Phenylalanine-Glutamic acid-Glutamine-Glutamine; SBF—Simulated Body Fluid; TKREEVD—Threonine-Lysine-Arginine-Glutamic acid-Glutamic acid-Valine-Aspartic acid.

## 3. Discussion

Enamel biomineralisation is a highly regulated process involving precise genetic control, as well as protein-protein interactions, protein-mineral interactions, and interactions involving the cell membrane. Ameloblast secretes enamel matrix proteins in the extracellular space between ameloblasts and dentine to control the initiation, orientation, nucleation, and growth of HAP crystals [4]. Amelogenin is the main secretory product of ameloblasts, making up more than 90% of the organic component in enamel [45]. The assembly of amelogenin has been shown to be crucial for the proper development of enamel crystallites. However, mature enamel is unable to heal and repair itself after demineralisation of the surface and subsequent cavitation. Biomimetic strategies for enamel regeneration may have the potential to repair enamel surface lesions. It is, therefore, important to study the structures and functions of amelogenin for the biomimetic mineralisation of enamel.

Iijima and Moradian-Oldak used the natural amelogenin *in vitro* to control calcium and phosphate crystallisation, and they found the growth of nanorod-like apatite crystals with a similar habit, size, and orientation to natural enamel [46]. Although this study was carried out through a cation selective membrane system as a model of tooth enamel formation, it highlighted the potential of amelogenin in the design and development of enamel-like biomaterials. Fan *et al.* used amelogenin with a modified biomimetic deposition method to remineralise the surface of etched enamel, and they were able to form mineral layers containing organised needle-like fluoridated hydroxyapatite crystals [28]. A follow-up study by Fan *et al.* reported the conditions of supersaturation degree, fluoride, and amelogenin concentration on the formation of densely packed fluoridated HAP on etched enamel *in vitro* [29]. An amelogenin-containing chitosan hydrogel was developed for superficial enamel reconstruction. The mechanism of the enamel reconstruction is through amelogenin supramolecular assembly, stabilising Ca-P clusters, and guiding their arrangement into linear chains [33,34]. However, it was difficult to extract and purify natural proteins. Thus, researchers fabricated protein analogues that could play the roles of the proteins in the biomineralisation process. Peptide/oligopeptide was fabricated to promote the formation of HAP crystals. A peptide with numerous repetitive nucleotide sequences of Aspartic-Serine-Serine (DSS), based on the sequence of dentin phosphoprotein had a high affinity to calcium phosphate compounds and promoted the formation of HAP crystals [39,40,42]. Triplet-repeating Asparagine-serine-serine (3NSS) peptide, a derivative of the DSS peptide, was designed by replacing the COOH group in Aspartic with the CONH_2_ group. The authors declared that enamel remineralisation with this 3NSS peptide showed a higher degree of recovery compared to DSS peptide. This might due to the difference between the ionic-attraction ability of a COOH group and CONH_2_ group [27,41,43]. Kirkham *et al.* designed a self-assembling peptide (P_11_-4, Ace-Gln-Gln-Arg-Phe-Glu-Trp-Glu-Phe-Glu-Gln-Gln-NH_2_), which could provide a biomimetic scaffold for HAP nucleation [31]. Recently, Li *et al.* fabricated an oligopeptide amphiphile (C_18_H_35_O-Thr-Lys-Arg-Glu-Glu-Val-Asp), which consisted of a derivative of stearic acid and a hydrophilic C-terminal amino residue of amelogenin [38]. In general, the peptides were shown to increase the remineralisation of enamel. PAMAM dendrimers have also been used as “artificial proteins” to evaluate their binding capacity and the surface charge with enamel crystals [12,47]. PAMAM-COOH acted as the organic template on the demineralized enamel surface to induce the formation of HAP crystals with the same structure, orientation and mineral phase of the natural enamel [30]. Due to the specific adsorption on HAP, alendronate was conjugated to PAMAM-COOH and induced *in situ* remineralisation of HAP on acid-etched enamel [36]. PAMAM-PO_3_H_2_ was designed with a similar dimensional scale and peripheral functions to that of amelogenin in enamel [37]. These dendrimers formed nanospheres and had a possible role in guiding crystal growth. In addition to the proteins and protein analogues, there are a number of other available biopolymers that were used in the biomimetic formation of HAP. These were predominantly polysaccharides such as agarose [19] and gelatine [20].

The synthesis of enamel-like structures based on the aforementioned approaches requires several days. In recent years, the biomimetic treatment of early caries lesions through the application of various types of bioactive materials with protein analogues has received considerable attention such as PAA-bioactive glass [32] and Glu-apatite nanoparticles [44]. PAA-bioactive glass was applied as slurry to avoid any damage to the enamel lesion structure. Bioactive glass is a ceramic material consisting of a similar amount of sodium, calcium, phosphate, and silicate ions, which are elements naturally found in the human body. The release of ions requires contact with artificial saliva. These bioactive materials dissolve in saliva to liberate ions that diffuse into the enamel subsurface lesion. PAA was used as an analogue to sequester calcium and phosphate ions released from bioactive glass to form amorphous calcium phosphate nano-precursors. The PAA-bioactive glass had the potential to deliver calcium and phosphate ions to subsurface enamel lesions. In an aqueous environment, the protonation of the Si-O groups formed rich silanols on the etched enamel surface. It generated an electronegative surface that provided heterogeneous nucleation sites for the formation of HAP crystals [48]. Amino acids are the basic building blocks of proteins. There is 15-20% Glu in the amino acid composition of amelogenin, and the roles of Glu in the biomimetic mineralisation of enamel have been investigated. Glu has two carboxylate groups that could be preferentially adsorbed onto the (001) face of apatite to induce the oriented crystallisation along the *c*-axis. Several studies demonstrated that apatite nano-particles have the potential to remineralise the initial enamel caries lesions *in vitro*. Nanostructured apatite crystals exhibit high levels of biomimetic properties and surface activity. Apatite nano-particles can act as a calcium and phosphate reservoir that helps to maintain a topical state of super-saturation of these ions on an enamel surface. As a building block, apatite nano-particles could adsorb onto the enamel surface and assemble into oriented HAP crystals under the control of Glu. This resulted in the regeneration of the enamel-like structure under physiological conditions [44].

In biology, the biomineralisation process is an organic matrix particle-mediated non-classical crystallisation pathway involving a mesoscopic transformation process. The presence of transient ACP nanoprecursors has been found during enamel biomineralisation [49]. The formation and stabilisation of amorphous nanoprecursors is an important step in the biomimetic mineralisation of enamel. CPP obtained from milk is an analogue of the proteins involved in the biomineralisation of teeth. CPP contains clusters of phosphorylated serine residues and can, thus, stabilise calcium and phosphate ions through the formation of amorphous nano-complexes [22,23,24]. CPP-ACP provided a reservoir of calcium and phosphate ions to maintain a state of super-saturation on the enamel surface, resulting in a significant remineralising effect on enamel subsurface lesions [50]. Based on the influence of phosphorylated proteins in enamel biomineralisation, nano-complexes of phosphorylated chitosan and ACP were synthesised to remineralise early enamel caries [25]. These results indicated that the application of natural protein analogues might be an effective strategy to stabilise the amorphous nanoprecursors.

Fluoride was used to prevent and arrest caries by inhibiting bacterial metabolism, inhibiting demineralisation, and enhancing remineralisation. Fluoride ions entered into the directive lattice of HAP crystals and replaced hydroxide ions or adsorbed onto the crystal surface to attract calcium and phosphate ions, resulting in the formation of fluoridated HAP [51]. Fluoridated HAP contains approximately 30,000 ppm fluoride and has a very low solubility in acid. In this review, fluoride was added in different remineralising mediums in 15 studies. The concentration of the fluoride used varied greatly between the studies. Fluoride had a significant effect on the morphology of calcium phosphate crystals. The morphology and orientation of the formed crystals were tuneable by the fluoride concentration. Iijima *et al.* discovered the conversion of ribbon-like octacalcium phosphate into needle-like HAP in a membrane system in the presence of 2 ppm fluoride [52]. Fan *et al.* reported that a critical fluoride at 5 ppm in remineralisation solutions with a pH of 6.74 and 5 mM Ca^2+^ was required to induce the transition from plate-like octacalcium phosphate to octacalcium phosphate rod-like HAP that regenerated on etched enamel surface [29]. The critical concentration of fluoride changed when the pH and the concentrations of Calcium and amelogenin were changed. Rod-like fluoridated HAP was reported in a hydrogel model with a high concentration of fluoride [19]. However, despite the predominant beneficial effect of fluoride on enamel remineralisation, the dental fluorosis induced by excessive intake of fluoride could not be ignored, especially with the application of these products to children.

Apparent progress has been made to understand the process of HAP biomineralisation of enamel, which has the potential to remineralise defective enamel lesions. A number of biomimetic mineralisation methods were reported and published in the literature. However, the overall mechanism of remineralisation *in vivo* has not yet been clarified. Further research is necessary to assess in detail whether the biomimetic mineralisation approaches for enamel caries therapy will be suitable and applicable in daily dental practice. Furthermore, an easy-to-apply, fast-growing system for enamel-like structure regeneration is necessary for clinical use.

## 4. Methods

Three electronic databases (PubMed, ScienceDirect, and ISI Web of Science) were systematically searched to identify publications before 1 January 2015. The keywords used were: ((biomimetic AND (mineralization OR mineralisation OR regeneration OR remineralization OR remineralisation)) OR biomineralization OR biomineralisation) AND enamel. No publication year or language limit was used. The titles and abstracts of identified articles were independently reviewed by two authors. Duplicated articles were removed. Clinical trials, reviews, non-English articles, animal teeth, non-tooth substrates, and irrelevant studies were excluded. The articles were further reviewed with full-texts if the articles met the inclusion criteria (biomimetic mineralisation methods for demineralised human enamel *in vitro*). Manual screening was conducted on the reference lists of all included articles to identify relevant articles. Disagreements about the inclusion or exclusion of a study were discussed with a third author for a final decision. Data extraction was conducted by one author from full-texts and evaluated by another one. Similar information mentioned in the included articles was summarised (such as protein and protein analogues).

## 5. Conclusions

Many studies with different methods reported success in the biomimetic mineralisation of enamel, including the use of protein and protein analogues, bioactive materials or components, an agarose hydrogel model, a glycerine-enriched gelatine technique, and ethylenediaminetetraacetic acid.
